# Sensors Information Fusion System with Fault Detection Based on Multi-Manifold Regularization Neighborhood Preserving Embedding

**DOI:** 10.3390/s19061440

**Published:** 2019-03-23

**Authors:** Jianping Wu, Bin Jiang, Hongtian Chen, Jianwei Liu

**Affiliations:** 1 College of Automation Engineering, Nanjing University of Aeronautics and Astronautics, Nanjing 211106, China; wujianping@nuaa.edu.cn (J.W.); chtbaylor@163.com (H.C.); ljw301@nuaa.edu.cn (J.L.); 2 Jiangsu Key Laboratory of Internet of Things and Control Technologies, Nanjing University of Aeronautics and Astronautics, Nanjing 211106, China

**Keywords:** sensor information fusion, locality preserving embedding (LPP), multi-manifold regularization neighborhood preserving embedding (MMRNPE), fault detection

## Abstract

Electrical drive systems play an increasingly important role in high-speed trains. The whole system is equipped with sensors that support complicated information fusion, which means the performance around this system ought to be monitored especially during incipient changes. In such situation, it is crucial to distinguish faulty state from observed normal state because of the dire consequences closed-loop faults might bring. In this research, an optimal neighborhood preserving embedding (NPE) method called multi-manifold regularization NPE (MMRNPE) is proposed to detect various faults in an electrical drive sensor information fusion system. By taking locality preserving embedding into account, the proposed methodology extends the united application of Euclidean distance of both designated points and paired points, which guarantees the access to both local and global sensor information. Meanwhile, this structure fuses several manifolds to extract their own features. In addition, parameters are allocated in diverse manifolds to seek an optimal combination of manifolds while entropy of information with parameters is also selected to avoid the overweight of single manifold. Moreover, an experimental test based on the platform was built to validate the MMRNPE approach and demonstrate the effectiveness of the fault detection. Results and observations show that the proposed MMRNPE offers a better fault detection representation in comparison with NPE.

## 1. Introduction

As a foundational component of industrial development, sensors of various types are applied and equipped in diverse systems [1,2], which perfectly meet the demand of data gathering [3,4] and fault detection [5,6,7]. In addition, the attractions of the information fusion process lie in its ability to eliminate the redundancy and contraction among sensor data sets, as well as its decision-making capacity with uncertainty information. However, once potential information gathered by sensors is not dealt with promptly, some tiny faults may extend unrestrictedly and result in breakdown of systems [7]. Therefore, for the sake of responsiveness, sensors information fusion is an essential technology for improving security and reliability of the system.

Several issues arise when fusing information is introduced to the system, including information uncertainty and data management. An efficient method to deal with the massive data information is feature extraction [8,9,10,11]. There are various techniques reported in the literature for fault diagnosis with the combination of algorithms’ fusion. Jafarian et al. [12] used fast Fourier transform as a feature extraction methodology, after which the artificial neural networks, support vector machines and k nearest neighbor classification algorithms are employed to verify the multiple performance metrics and realize signal monitoring. In addition, Saimurugan and Ramprasad [13] fused diverse algorithms for separate purposes to realize fault diagnosis. Wavelet transform and the decision tree were employed for feature extraction, in addition to the artificial neural network to classify the faulty situation. More recently, Liu et al. [14] proposed an intelligent multi-sensor data fusion method with the help of a relevance vector machine for gearboxes’ fault detection, and an ant colony optimization algorithm is involved.

However, the proposed compound methods are composed of diverse algorithms, which may result in the complexity of computational system for fault detection. For the purpose of detecting consistently and integrally, there should be some concise algorithms in data preprocessing, or one algorithm to realize the monitoring and detection of the whole system [15,16,17,18]. For example, Yunusa-Kaltungo et al. [19] proposed an improved composite spectrum data fusion technique to retain amplitude and phase information by applying cross power spectrum density to fault diagnosis in rotating machines. Moreover, Yunusa-Kaltungo et al. [20] used the data combination method as a preprocessing way for obtaining composite higher order spectra, after which principal component analysis was employed for fault detection. Jing et al. [21] employed deep convolutional neural networks to address an adaptive multi-sensor data fusion problem, which was capable of detecting the conditions of the planetary gearbox effectively with the best diagnosis accuracy. Most of the fusion strategies show their efficiency in small systems, whereas, in practical real-world scenarios, where data generated by sensors might be tremendous, these approaches might lose their advantages and lead to erroneous results.

In many critical fields, the systems are generally confronted with large scale and complicated logic, contributing to a high dimension of data collected from sensors. Under such circumstances, a manifold learning algorithm aiming at dimensionality reduction shows its advantage in data mining [22,23], such as neighborhood preserving embedding (NPE) [24], locality preserving projection (LPP) [25], Laplacian eigenmap (LE) [26], locally linear embedding (LLE) [27] and more [28,29,30,31]. It has been proved that the discriminative ability will be enhanced tremendously once the intrinsic manifold structure is considered. To be specific, NPE is a linear technology for combining neighbored data points together to seek an optimal local distribution. Nevertheless, NPE merely concerns the designated points, which means a lack of concern regarding the paired data.

At the same time, motivated by manifold learning algorithms, manifold regularized techniques, which also take local geometric structure into account, are proposed to learn a low-rank approximation [32,33]. For example, in [34], a structure cluster ensemble method is proposed to capture the structure information of the original data set with a manifold regularized objective function. In [35], Chuang et al. employ a manifold regularized distribution adaptation algorithm to classify both multi-spectral and hyper-spectral remote sensing data as well.

Inspired by the aforementioned research, this paper proposes a new algorithm named multi-manifold regularization neighborhood preserving embedding (MMRNPE). Different from the previous local manifold methods based on merely minimizing Euclidean distance between designated point and its neighbors, our framework pays extra attention to paired points in low dimension manifolds, along with the proportion adjustment between designated points and paired ones, which comprises the global information. It is also attractive because the selection of multi-manifold feature will avoid the disturbance and uncertainty of noise. Furthermore, multiple parameters are included for regularization or optimization purposes, which are capable of judging the membership between local and partial global message. In addition, as an iterable algorithm, MMRNPE is capable of choosing the iteration time by required accuracy but with consistent convergence.

The remainder of this paper is organized as follows. In Section 2, a brief review of some preliminaries and related works is given, including our small sensor information fusion system, as well as the NPE and LPP algorithms. In Section 3, the MMRNPE algorithm is proposed to extract both designated point information and paired points information, together with some discusions on algorithms and parameters. In Section 4, complete experiments based on the fusion system and MMRNPE are presented and verified. Conclusions are drawn in Section 5.

## 2. Background and Related Theoretical Reviews

### 2.1. One Small Sensor Information Fusion System

**Figure 1:**
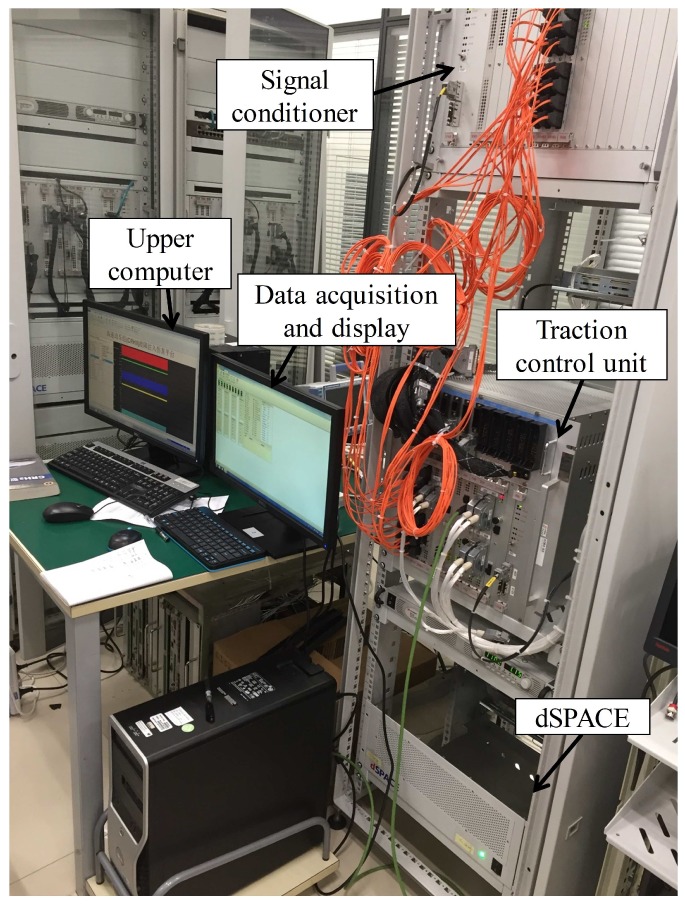
Small sensor information fusion system.

The small sensor information fusion system is actually an electrical drive system in high speed trains. Figure 1 presents the schematic diagram components of this platform [36]. It is an experimental platform of a high-speed train from the China Railway Rolling Stock Corporation in Zhuzhou, China. Several sensors are installed in different parts of traction components. By fusing the sensors and computational modules together with the aid of MATLAB R2014a (Mathworks, Natick, MA, USA) and dSPACE models (2014-A, dSPACE, Paderborn, Germany), the features of unexpected faults can be distinguished and the faults will be detected finally.

Effective sensor data is crucial to increase reliable detection capacity of this system. The more effective and complete the data set compiled from various sensors, the greater the system’s ability to extract features. In our electrical data-driven platform, three-phase output current signals collected from sensors equipped in traction motors are indicated as $i_{a},i_{b},i_{c}$. At the same time, the voltage from the line-side of a transformer is represented as $u_{net}$ while the two-phase input voltage signals of inverters are labeled as $u_{d1}$ and $u_{d2}$. The rotation signal of traction motor is *s*. In addition, sensors in traction inverters can also acquire boolean values as the judgement of switch state.

**Figure 2:**
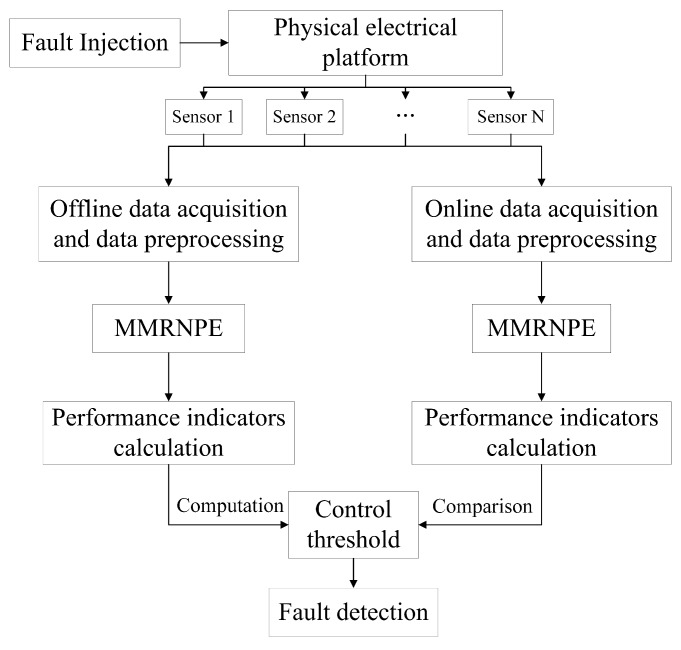
Construction of data-based information fusion structure.

Moreover, information fusion is a fundamental and essential part of sensor management. Since information from a single sensor may be inaccurate and uncertain, not only data collected from multiple sensors oughts to be fused in time, but also the acquired data in the inference and calculation process needs preprocessing. In addition, the following information fusion steps for analysis are based on the newly algorithm MMRNPE, which will be elaborated in Section 3. With the aid of the inference and calculation process, the control and management of the whole system will be realized once the monitoring of fault detection is enabled. The information fusion is, in fact, a data-based method. The process of information fusion structuring is shown in Figure 2.

To be specific, the offline data mainly consists of normal data sets, which means the calculated performance indicators represent the normal part. Once faults are injected into this physical electrical platform, the online data will change rapidly with the occurrence of jumping transition in performance indicators. With the aid of one accurate control threshold, the accuracy of faults detection might be guaranteed effectively. This sensor information fusion system is constructed for the purpose of fault detection. It fuses the information from multiple sensors to recognize abnormal signals through the proposed algorithm and make the right decisions accordingly.

### 2.2. Neighborhood Preserving Embedding

Neighborhood preserving embedding (NPE) is a recently proposed feature extraction method. The basic idea of NPE is to seek a lower dimensional projection of input sensor data set $X=\left[x_{1},x_{2},\ldots ,x_{N}\right]\in R^{D\times N}$.

To obtain the optimal projection matrix $A\in R^{N\times D}$, the NPE algorithm first constructs a neighborhood graph and then finds the weight matrix of each edge by minimizing the following reconstruction error [24,27]:

(1) $$\phi \left(W\right)=\min\sum_{i=1}^{N}∥x_{i}-\sum_{j=1}^{N}w_{ij}x_{j}{∥}^{2},$$

where $w_{ij}$ is the neighbored weight from $x_{i}$ to $x_{j}$, with constraints $\sum_{j=1}^{N}w_{ij}=1$ and $0\leq w_{ij}<1$. After acquiring the basic weight matrix, the minimized cost function with regard to the output matrix Y∈d×N(d<N) is then chosen as follows:

(2) $$\begin{array}{cc} \Phi (Y) & =\min_{yy^{T}=1}\sum_{i=1}^{N}∥y_{i}-\sum_{j=1}^{N}w_{ij}y_{j}{∥}_{2}^{2}\\ & =\min_{yy^{T}=1}a^{T}X{\left(I-W\right)}^{T}\left(I-W\right)X^{T}a\\ & =\min_{yy^{T}=1}a^{T}R_{NPE}a, \end{array}$$

where $R_{NPE}=X{\left(I-W\right)}^{T}\left(I-W\right)X^{T}$ and a is the column vector of matrix *A*. Now that $Y=A^{T}X$ is a linear projection, NPE is a linear approximate method that contributes to the fast computation ability among massive manifold learning algorithms. In addition, its focus on the relationship between the designated point and its neighbors is widely applied.

### 2.3. Locality Preserving Projection

The process of calculating the optimal weight matrix and reconstruction error of locality preserving projection (LPP) is the same as NPE. The only difference between these two algorithms is in their eigenmaps, i.e., the objective function of LPP is totally different from that of NPE.

(3) $$\begin{array}{cc} \Phi (Y) & =\frac{1}{2}\min_{yy^{T}=1}\sum_{i=1}^{N}\sum_{j=1}^{N}{\left(y_{i}-y_{j}\right)}^{2}w_{ij}\\ & =\frac{1}{2}\min_{yy^{T}=1}\sum_{i=1}^{N}\sum_{j=1}^{N}{\left(a^{T}x_{i}-a^{T}x_{j}\right)}^{2}w_{ij}\\ & =\min_{yy^{T}=1}\left(\sum_{i=1}^{N}a^{T}x_{i}D_{ii}x_{i}^{T}a-\sum_{i=1}^{N}\sum_{j=1}^{N}a^{T}x_{i}W_{ij}x_{j}^{T}a\right)\\ & =\min_{yy^{T}=1}a^{T}X\left(D-W\right)X^{T}a\\ & =\min_{yy^{T}=1}a^{T}XL_{LPP}X^{T}a, \end{array}$$

where *D* is a diagonal matrix with entries calculated from the column sum of W, i.e., $D_{ii}=\sum_{j}W_{ji}$. In addition, $L_{LPP}=D-W$ is a Laplacian matrix.

What makes LPP so attractive is its exploration towards finding another relationship between all paired points, that is, the variance based on the projected data with Euclidean distance. The detailed explanation is given in Section 3.

## 3. Multi-Manifold Regularization Neighborhood Preserving Embedding

As is mentioned in previous research [24,25], both NPE and LPP are linear manifold learning algorithms concentrating on extracting neighbored connections. In NPE, it reveals that NPE and LPP provide two different ways to linearly approximate the eigenfunction of Laplace Beltrom operator. However, in fact, they have totally different concerns that NPE focuses on the variance about the projected designated point and its reconstructed point while LPP concentrates on the variance about the projected paired points. In other words, NPE is more concerned about single data while LPP cares more about paired data. When it is projected into all data sets, NPE is a local algorithm and LPP is to some extent a global one.

Hence, this proposed multi-manifold regularization neighborhood preserving embedding is developed by combining them together to obtain an overall optimal algorithm. The detailed derivation of this MMRNPE algorithm is presented as below.

In LPP, $L_{LPP}=D-W$, where *W* is the weight matrix whose element $w_{ij}$ represents the neighborhood relationship between $x_{i}$ and $x_{j}$. However, in this MMRNPE, we replace *W* by *H*, where *H* is the matrix packaging the neighbored information:

(4) $$H_{ij}=\left\{\begin{array}{cc} 0, & \mathrm{if}\;x_{i}\;\mathrm{and}\;x_{j}\;\mathrm{are}\;\mathrm{neighbors},\\ 1, & \mathrm{if}\;x_{i}\;\mathrm{and}\;x_{j}\;\mathrm{are}\;\mathrm{not}\;\mathrm{neighbors}. \end{array}\right.$$

Actually, once the data set is given, two kinds of graph Laplacian can be established, including an unsupervised graph in which *L* is constructed by unlabeled data and a supervised graph in which *L* is constructed by labeled data. Unsupervised graph Laplacian is defined as ${\tilde{L}}^{\left(m\right)}=D^{\left(m\right)}-H^{\left(m\right)}$, where *m* is the number of manifolds. If labeled information is achievable, discriminative information is obtainable for separate samples of different labels. Therefore, a supervised graph Laplacian is constructed as ${\tilde{L}}^{\left(m\right)}={\tilde{L}}_{pos}^{\left(m\right)}-\beta {\tilde{L}}_{neg}^{\left(m\right)}$, where ${\tilde{L}}_{pos}^{\left(m\right)}$ and ${\tilde{L}}_{neg}^{\left(m\right)}$ represent normal data and faulty data, respectively. In addition, β is a regulating parameter. In this paper, a normalized graph Laplacian is proposed and applied as follows:

(5) $$L_{m}^{\left(i\right)}={D_{m}^{\left(i\right)}}^{-\frac{1}{2}}\left(D_{m}^{\left(i\right)}-H\right){D_{m}^{\left(i\right)}}^{-\frac{1}{2}},$$

where $D_{m}^{\left(i\right)}$ is the diagonal matrix similar to that in LPP and *i* here means the i-th manifold with particular setting. Thus, i=1,2,…,m.

However, there exists noise corruption in every manifold of $L_{m}^{\left(i\right)}$, which may result in the failure of exploring intrinsic distribution of samples and then the inaccuracy of fault detection. To avoid the accidental errors caused by a single manifold, one conjoint multi-manifold algorithm is proposed. The core of this multiple method is as follows:

(6) $$\begin{array}{c} L=\sum_{i=1}^{m}\alpha^{\left(i\right)}L_{m}^{\left(i\right)},\\ s.t.\;\sum_{i=1}^{m}\alpha^{\left(i\right)}=1, \end{array}$$

where $L_{m}^{\left(i\right)}$ are various manifolds that stem from different settings of neighborhood and $\alpha^{\left(i\right)}$ are the parameters to match the optimal multi-manifold combination. By expanding the choices of manifolds, the terminal selected *L* is in $L_{ter}$ = span{$L_{m}^{\left(1\right)},L_{m}^{\left(2\right)},\ldots ,L_{m}^{\left(m\right)}$}.

Actually, the idea of multi-manifold is confined to the introduction of $\alpha^{\left(i\right)}$, which is quite remarkable in our algorithm. Obviously, this approach is based on an assumption that the intrinsic manifold exactly lies in the convex hull of all the pre-given manifold candidates and these manifolds are the same as the concept of graph Laplacians. Thus, several manifolds corresponding to diverse neighborhood settings are gathered in the spanning set $L_{ter}$, which will ensure different features being collected and filtered. In addition, at the same time, the disturbance of noise and uncertainty from a single manifold are diminished.

Taking NPE and the above multi-manifold ideology from LPP into consideration, the objective function is changed:

(7) $$\begin{array}{cc} & \min\;a^{T}R_{\mathrm{NPE}}a-k\cdot a^{T}XLX^{T}a\\ = & \min\;a^{T}R_{\mathrm{NPE}}a-k\cdot \sum_{i=1}^{m}\alpha^{\left(i\right)}a^{T}XL_{m}^{\left(i\right)}X^{T}a\\ & s.t.\;\sum_{i=1}^{m}\alpha^{\left(i\right)}=1,\;a^{T}XX^{T}a=1, \end{array}$$

where $L=\sum_{i=1}^{m}\alpha^{\left(i\right)}\left({D_{m}^{\left(i\right)}}^{-\frac{1}{2}}\left(D_{m}^{\left(i\right)}-H\right){D_{m}^{\left(i\right)}}^{-\frac{1}{2}}\right)$ and *k* is a regularization parameter.

The parameter *k* here takes a role to scale the contribution of NPE and LPP. That is to say, although MMRNPE takes both local structure from NPE and the variance structure from LPP into consideration, there is no exact method to measure their own membership. Affiliated with the objected function, *k* adapts its role of adjusting the proportion of locally neighbored information and partial global variance information, which are critical to the distributive balance.

Use the Lagrange multiplier to solve the minimized problem, and the question is transformed into one generalized eigenvalue problem:

(8) $$\left[X^{T}MX-k\cdot XLX^{T}\right]a=\lambda XX^{T}a.$$

However, experiments indicate that the above objective function may concentrate on a series of problems with regard to manifold selection. To be specific, $\alpha =[\alpha_{1}\;\alpha_{2}\;\cdots \;\alpha_{m}]$ may meet with the following situation:

(9) $$\alpha_{i}=\left\{\begin{array}{cc} 0, & i\neq k,\\ 1, & i=k, \end{array}\right.$$

where *k* is the k-th manifold. This means that only the k-th parameter is efficient. In other words, although multiple manifolds are collected, only one particular manifold is chosen. Now that only one single manifold information is preserved and revealed, $L_{ter}$ is not used well. Hence, the objective function needs further improvement and constraint, which are about $\alpha^{\left(i\right)}$ to check and balance the decisive leadership of k-th manifold. Therefore, the task is to find a function $f(\alpha^{\left(i\right)})$ with negative correlation of $a^{T}XLX^{T}a$. Entropy of information [37,38,39] is an efficient method to make adjustment to the contribution on $\alpha^{\left(i\right)}$. Thus, another objective function is constructed to obtain $\alpha^{\left(i\right)}$:

(10) $$\begin{array}{c} \min\;a^{T}R_{\mathrm{NPE}}a-k\cdot \left[a^{T}XLX^{T}a+\gamma \sum_{i=1}^{m}\alpha_{i}\log\alpha_{i}\right],\\ s.t.\;\sum_{i=1}^{m}\alpha^{\left(i\right)}=1,\;a^{T}XX^{T}a=1, \end{array}$$

where γ is a parameter to adjust the proportion of entropy.

With the aid of the entropy of information about $\alpha^{\left(i\right)}$, the condition that one single manifold is emphasized obsessively will be avoided reasonably. With the introduction of γ, the cost function of $\alpha^{\left(i\right)}$ is modified. The existence of parameter γ along with the entropy of information is a penalty term of a generalized regularization, which does well in monitoring and regulating the validity of the objective function.

Therefore, the optimized objective function not only considers the local and partly global information, but also fuses multiple manifolds with sensor information. In addition, the added regularized parameters are also significant for selection of an optimal solution.

In a gesture to solve the minimization problem in Equation (10), Lagrange multipliers are introduced for constructing Lagrange function:

(11) $$\begin{array}{cc} Q= & \mathrm{tr}\left(a^{T}R_{\mathrm{NPE}}a\right)-k\cdot \sum_{i=1}^{m}\alpha^{\left(i\right)}\mathrm{tr}\left(a^{T}XL^{\left(i\right)}X^{T}a\right)\\ & -k\cdot \gamma \cdot \sum_{i=1}^{m}\alpha_{i}\log\alpha_{i}-\lambda_{1}\left(\sum_{i=1}^{m}\alpha^{\left(i\right)}-1\right)\\ & -\lambda_{2}\mathrm{tr}\left(a^{T}XX^{T}a-I\right), \end{array}$$

where $\lambda_{1}$ and $\lambda_{2}$ are Lagrange multipliers.

By setting the derivative of *Q* w.r.t. $\alpha^{\left(i\right)}$ along with $\lambda_{1}$ and $\lambda_{2}$ to zero, we have

(12) $$\begin{array}{c} \frac{\partial Q}{\partial \alpha^{\left(i\right)}}=-k\cdot \mathrm{tr}\left(a^{T}XL^{\left(i\right)}X^{T}a\right)-k\cdot \gamma \cdot \log\alpha_{i}-k\cdot \gamma -\lambda_{1},\\ \frac{\partial Q}{\partial \lambda_{1}}=-\sum_{i=1}^{m}\alpha^{\left(i\right)}+1,\\ \frac{\partial Q}{\partial \lambda_{2}}=-\mathrm{tr}\left(a^{T}XX^{T}a-I\right), \end{array}$$

so that we obtain $\alpha^{\left(i\right)}$ as follows:

(13) $$\alpha^{\left(i\right)}=\frac{\exp(\frac{-a^{T}XL^{\left(i\right)}Xa-\gamma }{\gamma })}{\sum_{i=1}^{m}\exp\left(\frac{-a^{T}XL^{\left(i\right)}Xa-\gamma }{\gamma }\right)}.$$

Now that $\alpha^{\left(i\right)}$ is deeply relevant to a, the variable $\alpha_{1}^{\left(i\right)}$ is initialized with a constant value, such as $\alpha_{1}^{\left(i\right)}=\left(1/m\right)$. Given $\alpha_{1}^{\left(i\right)}$, $a_{1}$ can be calculated from Equation (8). Then, a reliable $\alpha_{2}^{\left(i\right)}$ will be obtained from Equation (13). With the updated $\alpha_{2}^{\left(i\right)}$, *L* is completely new, and so are the manifolds. Thus, the eigenvectors of Equation (8) constitute $a_{2}$, which are the column vectors of projection matrix *A*. It is remarkable that the manifolds constructed by $\alpha_{2}^{\left(i\right)}$ are the expected results which are sensitive to suppressing the noise disturbance.

In accordance with the above analysis, the algorithmic procedure of the proposed MMRNPE can be formally summarized as below:

1. Compute the normalized graph Laplacians $L_{m}^{\left(i\right)}$ of different manifolds with Equation (5).
2. Compute the initial $L_{1}$ with pre-given manifold candidates:
  (14) $$L_{1}=\sum_{i=1}^{m}\alpha_{1}^{\left(i\right)}L_{m}^{\left(i\right)},\;\alpha_{1}^{\left(i\right)}=\frac{1}{m},$$
  where i=1,2,…,m.
3. Solve the generalized eigenvectors of the following equation as $a_{1}$:
  (15) $$\left[XMX^{T}-k\left(XL_{1}X^{T}\right)\right]a_{1}=\lambda XX^{T}a_{1},$$
  where $M={\left(I-W\right)}^{T}\left(I-W\right)$.
4. Compute $L_{2}$ with a series of optimized $\alpha_{2}^{\left(i\right)}$.
  (16) $$\begin{array}{cc} \alpha_{2}^{\left(i\right)} & =\frac{\exp(\frac{-a_{1}^{T}XL^{\left(i\right)}X^{T}a_{1}-\gamma }{\gamma })}{\sum_{i=1}^{m}\exp\left(\frac{-a_{1}^{T}XL^{\left(i\right)}X^{T}a_{1}-\gamma }{\gamma }\right)},\\ L_{2} & =\sum_{i=1}^{m}\alpha_{2}^{\left(i\right)}L_{m}^{i}. \end{array}$$
5. Solve the generalized eigenvectors of the following equation as $a_{2}$:
  (17) $$\left[XMX^{T}-k\left(XL_{2}X^{T}\right)\right]a_{2}=\lambda XX^{T}a_{2}.$$
6. Obtain the embedding as follows:
  (18) $$x_{i}\to y_{i}=A^{T}x_{i},\;A=\left[a_{2}^{\left(1\right)},a_{2}^{\left(2\right)},\ldots ,a_{2}^{\left(d\right)}\right].$$

**Figure 3:**
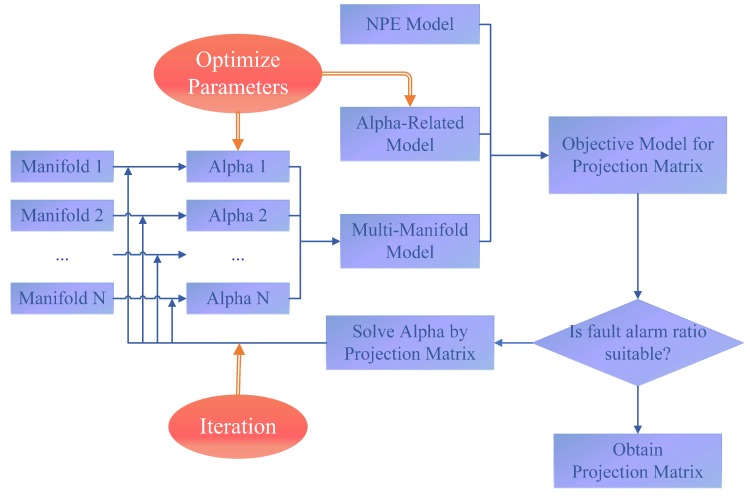
Iterable structure for MMRNPE.

Furthermore, the calculation process of our framework is an iterable one with the verification of regularization consistency shown in Figure 3.

The core of this process lies in the update of $\alpha^{\left(i\right)}$. More specifically, the iteration round will continue when $a_{2}$ in Equation (18) is substituted to Equation (13) and then $\alpha_{3}^{\left(i\right)}$ is obtained. After substituting $\alpha_{3}^{\left(i\right)}$ to Equation (8), it is easier to observe the projection matrix A. Such alternating iteration round is continuous and the convergency of this learning algorithm can always be guaranteed. Moreover, along with the increasing number of iterations, the algorithm is theoretically deeper, i.e., researchers may choose the iteration times freely and obtain the designing curve based on the requirement of experiment error plenarily.

The iteration procedure is an optimal process of parameter $\alpha^{\left(i\right)}$ as well.

In general, MMRNPE is proved to be a successful method in exploiting the underlying geometry structure of selected data sets with the aid of NPE and LPP. Actually, by incorporating the ideology of LPP or graph Laplacians, MMRNPE takes advantage of NPE’s local structure and LPP’s partial global variance structure successfully. It is worthwhile to highlight the marvelous properties of this proposed approach:

- In our MMRNPE, it takes the Euclidean distance of both designated point and paired points into adequate consideration, which guarantees the balance between local and global information from sensor data.
- Multiple parameters are included in this algorithm, some of which are of regularization purpose and the others are of limitation consideration. Now that various choices of parameters will result in distinction of performance, some optimization algorithms can be chosen to promise the fault detection rate.
- Some of the regularized parameters are able to judge the membership relationship of elements, i.e., the membership between local information with NPE algorithm and variance information with LPP algorithm is displayed intuitively, which realizes the sensor information fusion.

## 4. Experiments

### 4.1. Fault Detection Strategy

The fault detection is based on the small sensor information fusion system referred to in Section 2.1. The detailed parameters information is given in Table 1 [7], which includes parameters both in physical space and in computational space.

Actually, data sets collected from sensors in physical space are sent to the computational space promptly, after which the calculation and monitoring process begins. The calculation and monitoring process includes two steps—offline calculation and online monitoring.

Now that MMRNPE seeks a latent variable space that represents the high dimensional space relatively, the monitoring statistics Hotelling’s $T^{2}$ statistic is constructed as a measurement of the performance of fault detection [40,41]. Here is the definition of $T^{2}$:

(19) $$\begin{array}{cc} T^{2} & =X^{T}A\Lambda^{-1}A^{T}X\\ & =X^{T}A{\left(\mathrm{cov}\left(Y_{\mathrm{offline}}\right)\right)}^{-1}A^{T}X\\ & =Y^{T}{\left(\mathrm{cov}\left(Y_{\mathrm{offline}}\right)\right)}^{-1}Y\\ & =\frac{1}{n-1}Y^{T}Y_{\mathrm{offline}}^{T}Y_{\mathrm{offline}}Y, \end{array}$$

where $\Lambda =\mathrm{cov}(Y_{\mathrm{offline}})$ is the covariance matrix of the offline data set and *Y* is the sample after dimensional reduction process. It is obvious that with sampling n, $T^{2}$ is the sum of the normalized squared scores, making it possible to measure the performance of the chosen projection matrix A [42,43]. Another statistic SPE plays this role as well [40,41].

With the performance statistics obtained, the offline modeling procedure is as follows:

1. Collect original data set X and normalize it with zero mean and unit variance.
2. Compute the projection matrix with the proposed MMRNPE algorithm.
3. Calculate the dimensional-reduction data set Y with the linear mapping.
4. Compute the performance statistics $T^{2}$ and SPE of offline data set.
5. Construct the upper control limits of $T^{2}$ and SPE as the standard of online data.

After the offline modeling process, the upper control limits are obtained, and then we can implement the online monitoring procedure:

1. Collect online data set $X_{\mathrm{online}}$ and normalize it with zero mean and unit variance.
2. Calculate the dimensional-reduction data set Y with the projection matrix obtained in offline procedure.
3. Compute the performance statistics $T^{2}$ and SPE of the online data set and compare them with the upper control limits of $T^{2}$ and SPE from the offline process.
4. Compute the fault alarm ratio (FAR), non-detection ratio (NDR) and total detection rate (TDR) to evaluate the fault detection ability of this MMRNPE algorithm.

### 4.2. Experiments Verification with the Proposed MMRNPE

The platform with multiple sensors to fuse information derived in Section 2.1 is selected to verify this multi-manifold algorithm.

Several experiments are carried out in the normal state and various bias faulty states on the test bench. Both the normal and faulty expressions are listed in Table 2.

Numerous sensors are equipped and distributed across all aspects and locations of the system. Several typical signals attract much attention to evaluate the performances of motors, including current signals, voltage signals and speed signals. Hence, we locate the sensor faults at current path and voltage path, as well as the speed sensor itself separately. As is obviously shown in Table 2, three types of faults are manually induced in various sensors under different operation conditions, i.e., current sensor fault, voltage sensor fault and speed sensor fault.

**Figure 4:**
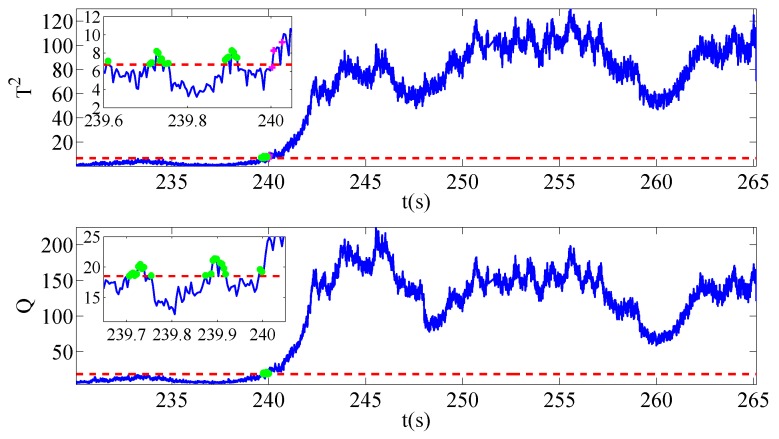
Fault detection results for $f_{1}$.

**Figure 5:**
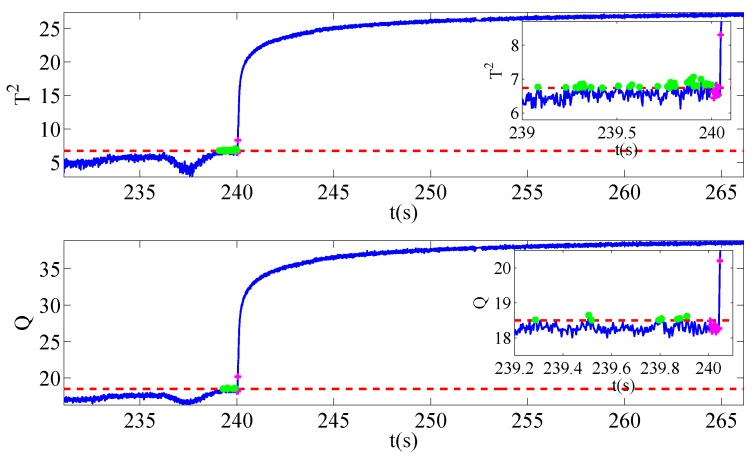
Fault detection results for $f_{2}$.

**Figure 6:**
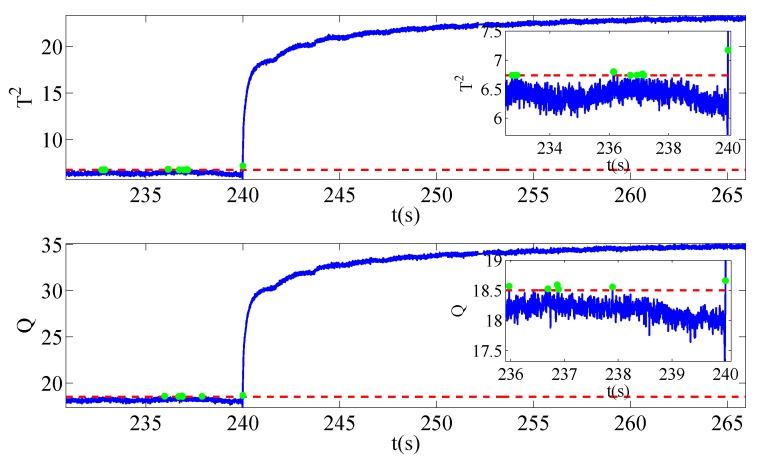
Fault detection results for $f_{3}$.

Once sensors located in other locations break down and affect the security of system operation, the typical signals mentioned above also changes abnormally. In a gesture to evaluate the severities of faults, three different ranks of signal amplitude are set. The degree of voltage sensor fault is 0.01% of the running voltage amplitude while that of current sensor fault is 0.05%. In addition, the speed sensor fault is 0.5% of the normal condition. It should also be noted that the training data of three different faults share the same normal data sets. The only difference lies in the test data, where samplings collected during the faulty operation periods have the same length. Figure 4, Figure 5 and Figure 6 give the evolution processes of fault injections with different sensor faults.

The sensor faults are injected into the platform at 240 s. With the aid of this MMRNPE structure proposed for fault detection, three faults are detected successfully in our experiments.

The parameters set in this experiment are chosen as below. Three manifolds are constructed with $\alpha_{1}^{\left(1\right)}=\alpha_{1}^{\left(2\right)}=\alpha_{1}^{\left(3\right)}=\frac{1}{2}$. By choosing k=8 and γ=5689.9, $\alpha_{2}^{\left(i\right)}$ can be calculated that $\alpha_{2}^{\left(1\right)}=0.3826$, $\alpha_{2}^{\left(2\right)}=0.3375$, and $\alpha_{2}^{\left(3\right)}=0.2799$.

As is shown in Figure 4, Figure 5 and Figure 6, there are some fault alarming points which are below control limits after faults occur and non-detection points that are above control limits before faults happen. Both fault alarming points and non-detection points are marked in the figures with striking colors. At the same time, fault alarming ratio (FAR), non-detection ratio (NDR) and total detection ratio (TDR) with performance statistics $T^{2}$ and SPE are calculated under both MMRNPE and NPE algorithms, which are shown in Table 3.

The detection indexes of various sensor faults with MMRNPE and NPE shown in Table 3 can obviously verify the superiority of our MMRNPE, especially when it comes to statistics $T^{2}$. The results of FAR, NDR and TDR with performance statistics $T^{2}$ perform better accuracy of fault detection. Furthermore, with a careful observation of the magnified figures, it is apparent that several misclassifications occur. To be more specific, the non-detection point may be marked in the fault alarming notation after the fault is injected while the fault alarming point may be marked in a non-detection notation before 240 s. Such mistakes take place due to the setting of sampling time. The sampling time $T_{s}$ is $4\times 10^{-4}$ s, which will make it difficult to inject the sensor faults exactly at 240 s, i.e., the injected time 240 s is between sampling points m and m+1.

**Figure 7:**
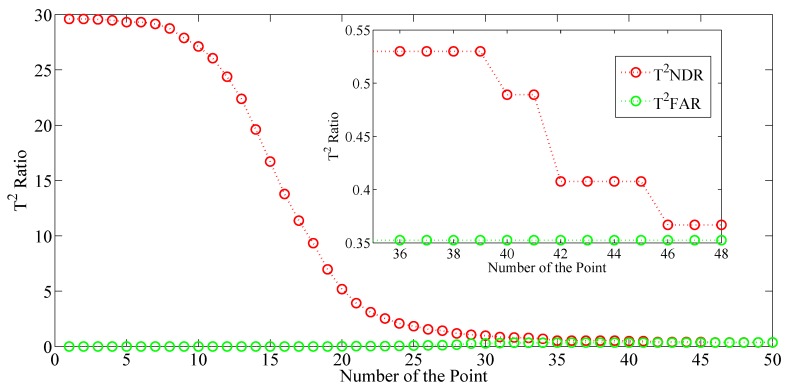
Fault detection results for $f_{3}$.

Several parameters are induced into this MMRNPE algorithm and each of them plays its own role. Here, we will discuss the role of γ to select an optimal one. By using different γ ,the monitoring and detection results of $f_{1}$ with MMRNPE are totally different, which are shown in Figure 7.

As is shown in Figure 7, the FAR of $T^{2}$ increases very slightly while NDR decreases like an inverse sigmoid curve with the gradual increase of γ.

The *x*-axis of Figure 7 is the number of γ, where the series of γ is actually a geometrical sequence with the initial value is 1 and the terminal value is $10^{4}$.

## 5. Conclusions

Multiple sensors located at various positions of this electrical drive system are comprised of numerous amounts of characteristic information. In this research, we discussed the excavation of sensor data sets obtained from sensors information fusion systems to detect faults via an adapted MMRNPE algorithm. There are three key components of this improved algorithm. Firstly, as a combination of NPE and LPP, the objective function of MMRNPE inspired by manifold learning algorithms considers both the designated points and paired points to find the intrinsic incorporation. Secondly, multiple manifolds with various neighborhood points are merged together closely while keeping their distinct characteristics. Thirdly, diverse parameters are introduced and presented into this methodology to play their own role, some of which bear the responsibility of adjusting the proportion of locally neighbored information and partial global variance information. In addition, some of the parameters are used for weighting adjustments with different manifolds. The experimental results demonstrate that MMRNPE realizes data processing and information fusion successfully in terms of its sufficient fault detection effects. With three different sensor faults injected and detected promptly and efficiently, this approach is verified and confirmed adequately.

In this study, parameters are selected in an enumeration or experimental way, which means future work will focus on the optimization algorithms for our strategy. By taking the optimization algorithms as well as iteration into account, the detection efficiency will be promoted to a higher level.

## Figures and Tables

**Table 1:** The parameters for the platform.

Symbol	Quantity	Value (Unit)
$T_{s}$	sampling time	$4\times 10^{-4}$ (s)
*p*	pole pairs	2(1)
$U_{s}$	voltage of charge source	400 (V)
$R_{s}$	resistance in stator side	0.228(Ω)
$R_{r}$	resistance in rotor side	0.1267(Ω)
$L_{s}$	inductance in stator side	0.0281 (H)
$L_{r}$	inductance in rotor side	0.0280 (H)
$L_{msr}$	mutual inductance of motor	0.0268 (H)
$L_{ls}$	leakage inductance in stator side	0.0013 (H)
$L_{lr}$	leakage inductance in rotor side	0.0013 (H)
$U_{d}$	intermediate voltage	3300 (V)
$C_{d}$	capacitor of direct current link	$8\times 10^{-3}$ (F)
*J*	rotary inertia	100 (kg·m${}^{2}$)
*L*	filter inductance	$0.42\times 10^{-3}$ (H)
*C*	filter capacitor	$6\times 10^{-3}$ (F)

**Table 2:** The parameters for concerned states.

Notations	Fault Description	Expression	Sample Number
*N*	normal	normal	250
$F_{1}$	current sensor fault	$f_{1}=0.05$ A	8695
$F_{2}$	voltage sensor fault	$f_{2}=2$ V	8695
$F_{3}$	speed sensor fault	$f_{3}=0.5$ rad/s	8695

**Table 3:** The detection indexes of various sensor faults with MMRNPE and NPE.

Algorithm		MMRNPE			NPE		
Sensor Fault	Statistics	FAR(%)	NDR(%)	TDR(%)	FAR(%)	NDR(%)	TDR(%)
$f_{1}$	SPE	0.8464	0	0.0024	0.8464	0	0.0024
	$T^{2}$	0.6449	0.0322	0.0021	0	12.3270	0.0881
$f_{2}$	SPE	0.3136	0.1393	0.0021	0.4032	0.1547	0.0022
	$T^{2}$	1.4337	0.1238	0.0048	11.0663	0.0464	0.0288
$f_{3}$	SPE	0.1754	0	0.000345	0.2630	0	0.0069
	$T^{2}$	0.2630	0	0.0010	0.3725	0.0156	0.0015

## Abbreviations

The following abbreviations are used in this manuscript:

NPE	Neighborhood Preserving Embedding
MMRNPE	Multi-Manifold Regularization Neighborhood Preserving Embedding
LPP	Locality Preserving Embedding
LE	Laplacian Eigenmap
LLE	Locally Linear Embedding
SPE	Square Prediction Error
FAR	Fault Alarm Ratio
NDR	Non-Detection Ratio
TDR	Total Detection Ratio
